# Author Correction: Molecular estimation of neurodegeneration pseudotime in older brains

**DOI:** 10.1038/s41467-020-20261-6

**Published:** 2020-12-03

**Authors:** Sumit Mukherjee, Laura Heath, Christoph Preuss, Suman Jayadev, Gwenn A. Garden, Anna K. Greenwood, Solveig K. Sieberts, Philip L. De Jager, Nilüfer Ertekin-Taner, Gregory W. Carter, Lara M. Mangravite, Benjamin A. Logsdon

**Affiliations:** 1 Sage Bionetworks, Seattle, WA USA; 2 The Jackson Laboratory, Bar Harbor, ME USA; 3 Department of Neurology, University of Washington, Seattle, WA USA; 4 Center for Translational & Computational Neuroimmunology, Department of Neurology, Columbia University Irving Medical Center, New York City, NY USA; 5 Taub Institute, Columbia University Irving Medical Center, New York City, NY USA; 6 Department of Neurology, Mayo Clinic Florid, Jacksonville, FL USA; 7 Department of Neuroscience, Mayo Clinic Florida, Jacksonville, FL USA; 8 Present Address: Microsoft, Redmond, WA USA; 9 Present Address: Cajal Neuroscience, Seattle, WA USA

**Keywords:** Machine learning, Gene expression, Dementia

Correction to: *Nature Communications* 10.1038/s41467-020-19622-y, published online 13 November 2020.

In the original version of this Article, the Data Availability statement was incomplete. The following text has been added to the Data Availability statement: ‘The data described in this manuscript are available via the AD Knowledge Portal (https://adknowledgeportal.org). The AD Knowledge Portal is a platform for accessing data, analyses, and tools generated by the Accelerating Medicines Partnership (AMP-AD) Target Discovery Program and other National Institute on Aging (NIA)-supported programs to enable open-science practices and accelerate translational learning. The data, analyses, and tools are shared early in the research cycle without a publication embargo on secondary use. Data are available for general research use according to the following requirements for data access and data attribution (https://adknowledgeportal.synapse.org/DataAccess/Instructions). See: 10.7303/syn23262875.’

